# Optimization of d-lactic acid production using unutilized biomass as substrates by multiple parallel fermentation

**DOI:** 10.1007/s13205-016-0499-2

**Published:** 2016-08-31

**Authors:** Elya Mufidah, Mamoru Wakayama

**Affiliations:** Department of Biotechnology, Graduate School of Life Sciences, Ritsumeikan University, 1-1-1 Nojihigashi, Kusatsu, Shiga 525-8577 Japan

**Keywords:** d-Lactic acid, *Leuconostoc mesenteroides*, Banana peel, Corncob, Multiple parallel fermentation, Optimization

## Abstract

This study investigated the optimization of d-lactic acid production from unutilized biomass, specifically banana peel and corncob by multiple parallel fermentation (MPF) with *Leuconostoc mesenteroides* and *Aspergillus awamori*. The factors involved in MPF that were assessed in this study comprised banana peel and corncob, KH_2_PO_4_, Tween 80, MgSO_4_·7H_2_O, NaCl, yeast extract, and diammonium hydrogen citrate to identify the optimal concentration for d-lactic acid production. Optimization of these component factors was performed using the Taguchi method with an L8 orthogonal array. The optimal concentrations for the effectiveness of MPF using biomass substrates were as follows: (1) banana peel, d-lactic acid production was 31.8 g/L in medium containing 15 % carbon source, 0.5 % KH_2_PO_4_, 0.1 % Tween 80, 0.05 % MgSO_4_·7H_2_O, 0.05 % NaCl, 1.5 % yeast extract, and 0.2 % diammonium hydrogen citrate. (2) corncob, d-lactic acid production was 38.3 g/L in medium containing 15 % of a carbon source, 0.5 % KH_2_PO_4_, 0.1 % Tween 80, 0.05 % MgSO_4_·7H_2_O, 0.1 % NaCl, 1.0 % yeast extract, and 0.4 % diammonium hydrogen citrate. Thus, both banana peel and corncob are unutilized potential resources for d-lactic acid production. These results indicate that MPF using *L. mesenteroides* and *A. awamori* could constitute part of a potential industrial application of the currently unutilized banana peel and corncob biomass for d-lactic acid production.

## Introduction

Lactic acid plays significant roles in several biochemical processes and exists in nature as two optical isomers, d- and l-lactic acid (Benninga 1990). Lactic acid is found primarily in sour milk products, such as *koumiss*, yogurt, kefir, and some cottage cheeses. The casein in fermented milk is coagulated (curdled) by lactic acid. Lactic acid is also responsible for the sour flavor of sourdough breads (Carminati et al. 2010). In the chemical industry, both d- and l-lactic acid have many uses as food chemicals, preservatives, oxygenated chemicals, plant growth regulators, raw materials for biodegradable polymers and environmentally friendly solvents (Datta and Tsai 1997; Richard and Nwabunma 2010; Wang et al. 2003).

Various microorganisms produce sufficient amount of lactic acid using various sugars, such as glucose, sucrose, and lactose (Richard and Nwabunma 2010). For example, glucose can be easily exploited to produce 25.4 g/L of l-lactic acid in LB medium after 48 h through simple fermentation using *Bacillus licheniformis* (Wang et al. 2011). It was also reported that *Leuconostoc mesenteroides* produced 66.11 g/L of d-lactic acid using an optimal combination of yeast auto-lysate and sugarcane juice containing sucrose (Coelho et al. 2011) and recombinant *E. coli* JH15 produced 88 g/L of d-lactic acid from a mixed glucose and xylose in 36 h fermentation (Lu et al. 2016). Similarly, *Rhizopus oryzae* ATCC 52311 produced 83 g/L of l-lactic acid from glucose in 32 h of fermentation (Zhou et al. 1999).

Agricultural residues such as banana peel and corncob are potential sources of renewable energy (Rehman et al. 2014; Jeevan et al. 2011). These unutilized raw biomaterials have also received attention as potential substrates for lactic acid fermentation. When unutilized biomass is used as a carbon source, it must first be saccharified because most lactic acid bacteria cannot utilize it directly (Luo et al. 1997; Ghowdaman and Ponnusami 2015). In addition, pretreatment such as alkali or acid treatment of unutilized raw biomaterials is often required before saccharification. Pretreatment methods for unutilized raw biomaterials have an influence on saccharification efficiency and will indirectly affect for lactic acid production (Sasaki et al. 2012). There have been reports on lactic acid production using unutilized raw biomaterials. Using corncob as a substrate, *R. oryzae* NRRL-395 produced 299.4 g l-lactic acid per kg dry matter in 48 h of fermentation at 30 °C (Ruengruglikit and Hang 2003), while simultaneous saccharification and fermentation of *Acremonium cellulose* and *Rhizopus* sp. produced 24.0 g/L l-lactic acid in 48 h (Miura et al. 2004). Additionally, *Lactobacillus delbrueckii* IFO 3202 produced 28 kg m^−3^ d-lactic acid using 100 kg m^−3^ rice bran as a substrate in 36 h at 37 °C by simultaneous saccharification and fermentation (Tanaka et al. 2005) and *L. delbrueckii* produced 16.5 g/L of d-lactic acid using sugarcane bagasse and a steam explosion pretreatment (Sasaki et al. 2012).

As described above, d-lactic acid production by *L. mesenteroides* using sugarcane juice has been reported (Coelho et al. 2011). Although sugarcane juice is easily exploited as a base material for d-lactic acid fermentation without a time- and labor-consuming pretreatment, sugarcane is also valuable, primarily as sweetener in the food industry. Thus, production of d-lactic acid from fermentation of otherwise unutilized raw biomaterials, such as banana peel, corncob, rice bran, and sugarcane bagasse, would be advantageous. There have been no reports on d-lactic acid production from banana peel or corncob by *L. mesenteroides.* In this case, a pretreatment with safe chemical reagents on unutilized biomass substrate, banana peel and corncob should be taken into account. Consequently, it leads to an efficient hydrolysis of biomass substrates by fungi, which combines with d-lactic acid fermentation process using *L. mesenteroides*. The present study investigated optimization of d-lactic acid production using banana peel and corncob as fermentation substrates for multiple parallel fermentation via *Leuconostoc mesenteroides* and *Aspergillus awamori*, a *koji* mold used in Japanese spirit brewing.

## Materials and methods

### Microorganisms

The lactic acid bacteria used in this study were *Leuconostoc mesenteroides* subsp. *mesenteroides* (NBRC 3426, NBRC 3832, and NBRC 12060), and *Leuconostoc mesenteroides* subsp. *dextranicum* (NBRC 3349, NBRC 100495). The following fungi were evaluated for their capacity to aid in degradation of biomass substrates, *Aspergillus niger* NBRC 4414, *Aspergillus awamori* NBRC 4388*, Rhizopus oryzae* NBRC 4706, *Rhizopus microspores* NBRC 3200*, Aspergillus oryzae* NBRC 100959, and *Aspergillus sojae* NBRC 33802.

### Media and cultivation

#### Medium condition for fungi

For fungi cultivation, the growth liquid medium was 1 % of a carbon source (banana peel or corncob), 0.25 % KH_2_PO_4_, 0.1 % yeast extract, 0.035 % MgSO_4_·7H_2_O, 0.035 % urea, 0.1 % Tween 80, 0.005 % FeSO_4_·H_2_O, 0.001 % MnSO_4_·H_2_O, and 0.1 % NaCl. The initial pH was adjusted to 5.6. A total of 5 mL of the inoculum with 3 % of calcium carbonate was transferred to 100-mL Erlenmeyer flasks containing 50 mL of medium. Cultivation was carried out in flask cultures at 30 °C on a rotary shaker at 120 rpm.

#### Medium condition for lactic acid bacteria

MRS medium was used for lactic acid bacteria (Coelho et al. 2011). The liquid MRS composition was as follows: 1.5 % poly peptone, 0.5 % yeast extract, 1.0 % beef extract, 2.0 % glucose, 0.7 % sodium acetate, 0.3 % diammonium hydrogen citrate, 0.4 % di-potassium hydrogen phosphate, 0.02 % MgSO_4_·7H_2_O, and 0.004 % MnSO_4_·4H_2_O. The initial pH was adjusted to 6.3. A total of 10 mL of the inoculum with 3 % of calcium carbonate was transferred to a 200-mL Erlenmeyer flask containing 100 mL of the medium. Cultivation was carried out in flask cultures at 30 °C on a rotary shaker at 100 rpm.

### Pretreatment of fermentation substrates

Banana peel (*Musa acuminata balbisiana Colla*) was collected from household waste, and corncob (*Zea mays*) was supplied by a small cornstarch factory in Bali, Indonesia. Before banana peel was utilized, it was cut into small 5- to 8-cm pieces using a stainless steel knife. The banana peel was then soaked in 1 % sodium thiosulfate solution for 5 h at 30 °C to inhibit the oxidation processes, thus preventing browning to get a better texture for use of the banana peel in lactic acid production (unpublished data). Milled corncob was soaked in a 0.5 % sodium hypochlorite solution for delignification for approximately 6 h at 30 °C, followed by a drying process in a hot air oven (Ghowdaman and Ponnusami 2015). The small dried pieces of both banana peel and corncob were collected by sieving with a <1-mm mesh net.

### Multiple parallel fermentation

Multiple parallel fermentation (MPF) is the combination of biomass substrate degradation by fungi with lactic acid fermentation by *L. mesenteroides*. After cultivation of fungi for 36 h, *L. mesenteroides* NBRC 3832 was inoculated to the culture medium of fungi for d-lactic acid production by MPF. The liquid medium for multiple parallel fermentation included 3 % carbon source (banana peel or corncob), 0.25 % KH_2_PO_4_, 0.5 % yeast extract, 0.05 % MgSO_4_·7H_2_O, 0.05 % urea, 0.1 % Tween 80, 0.005 % FeSO_4_·H_2_O, 0.001 % MnSO_4_·H_2_O, 0.2 % diammonium hydrogen citrate and 0.1 % NaCl. The initial pH was adjusted to 5.6. A total of 10 mL of the inoculum with 3 % calcium carbonate was transferred to a 300-mL Erlenmeyer flask containing 200 mL of medium. MPF was performed at 30 °C in an incubator with gentle shaking at 120 rpm during hydrolysis by fungi and at 100 rpm after hydrolysis to allow the media and inoculum to be mixed homogeneously.

### Analytical methods

Reducing sugar was measured using the Somogyi–Nelson method (Somogyi 1952). The protein concentration was determined using either the Lowry method with bovine serum albumin as the standard (Lowry et al. 1951) or by monitoring the optical density at 280 nm. The activities of xylanase, pectinase, amylase, β-glucosidase, and endoglucanases in culture mediums with banana peel and corncob as biomass substrates were determined as follows. Crude enzyme preparation (100 μL) was incubated with the relevant substrate (1 % w/v) (beech wood xylan for xylanase, citrus pectin for pectinase, starch for amylase, salicin for β-glucosidase, and carboxymethyl cellulose for endoglucanase) in 50 mM acetate buffer at pH 5.0 (1.0 mL of reaction mixture). The amount of reducing sugars was determined using the Somogyi–Nelson method against the standard curves of xylose, galacturonic acid, and glucose. The amount of enzyme that released 1 μmol of reducing sugar per minute under standard assay conditions was defined as one international unit (U) of enzyme. d-Lactic acid was measured according to the instructions from Boehringer Mannheim/R-Biopharm with minor modification. The reaction solution consisted of 222 mM glycylglycine buffer (pH 10.0), 100 mM l-glutamic acid, 52.8 mM NAD^+^, 13.66 U of glutamate–pyruvate transaminase, and 6.79 U of d-lactate dehydrogenase. The absorbance increase of NADH at 340 nm was determined. Measurement of l-lactic acid was also carried out using 43.48 U of l-lactate dehydrogenase instead of d-lactate dehydrogenase.

### Experimental design

The optimal MPF conditions for production of d-lactic acid were determined using the method from Taguchi, in which variables or factors were arranged in an L8 orthogonal array (OA). All calculations and analyses were performed using Qualitek-4 software for automatic design and analysis of Taguchi experiments. The factor levels and L8 OA layout are presented in Table 1a and b. The signal-to-noise ratio (S/N) shows the extent of all factor effects. This approach introduces the S/N ratio to examine the influence of the noise factor on variation. For the S/N ratio, characteristic types defined by the larger the better means that the highest value is the best quality (Ravella et al. 2008).

**Table 1:** Condition of factors and its level (a) and experimental design using L_8_ (2^7^) orthogonal array (b) of Taguchi method for multiple parallel fermentation using banana peel and corncob as carbon sources

(a) Factors	Level 1	Level 2
X1. Banana peel (a), corncob (b); (%)	10	15
X2. KH_2_PO_4_ (%)	0.5	1
X3. NaCl (%)	0.05	0.1
X4. Tween 80 (%)	0.05	0.1
X5. Yeast extract (%)	1	1.5
X6. MgSO_4_·7H_2_O (%)	0.05	0.1
X7. Diammonium hydrogen citrate (%)	0.2	0.1

(b) Run	Factors							SNR of LTB^a^	SNR of LTB^b^
	X1	X2	X3	X4	X5	X6	X7		
1	1	1	1	1	1	1	1	22.812 ± 0.003	25.713 ± 0.021
2	1	1	1	2	2	2	2	22.088 ± 0.023	25.555 ± 0.004
3	1	2	2	1	1	2	2	19.647 ± 0.007	25.342 ± 0.004
4	1	2	2	2	2	1	1	22.524 ± 0.011	25.660 ± 0.022
5	2	1	2	1	2	1	2	29.530 ± 0.013	29.115 ± 0.032
6	2	1	2	2	1	2	1	29.011 ± 0.022	29.327 ± 0.041
7	2	2	1	1	2	2	1	28.708 ± 0.033	25.889 ± 0.007
8	2	2	1	2	1	1	2	29.230 ± 0.014	31.233 ± 0.004

The number (1 and 2) below each factor (X1–X7) indicates the level of each factor described in Table 1b

The values of signal-to-noise ratio (S/N ratio) represent the mean ± SD (*n* = 3)

In signal-to-noise ratio, characteristic types of LTB (larger the better) means the highest value is the best of quality (Ravella et al. 2008)

The factors and their values were determined based on triplicate experiments (*n* = 3)

*X1–X7* are name of each factor, *XI*: carbon source; ^a^banana peel concentration; ^b^corncob concentration, *X2* KH_2_PO_4_, *X3* NaCl, *X4* Tween 80, *X5* yeast extract, *X6* MgSO_4_·7H_2_O, *X7* diammonium hydrogen citrate

## Results and discussion

### Screening of microorganisms

The liquid fermentation abilities of the microorganisms used in this study were assessed to determine the bacteria that produced the highest amounts of d-lactic acid. Among the lactic acid bacteria examined, *L. mesenteroides* NBRC 3832 produced the highest amounts of d-lactic acid (6.74 g/L) by simple fermentation using 2 % glucose in MRS medium, with sugar consumption of approximately 89.1 % during 96 h (unpublished data). Next, to select the fungus that is most suitable for MPF with *L. mesenteroides* NBRC 3832, fungal contribution to d-lactic acid production via the saccharification of biomass substrates was evaluated. *A. niger* NBRC 4414, *A. awamori* NBRC 4388, *R. oryzae* NBRC 4706, *R. microspores* NBRC 3200, *A. oryzae* NBRC 100959, and *A. sojae* NBRC33802 were tested in a preliminary experiment. Of these, using banana peel and corncob, *A. awamori* NBRC 4388 showed the highest d-lactic acid production by MPF with *L. mesenteroides* NBRC 3832. In this case, *A. awamori* NBRC 4388 was cultured in the liquid medium for MPF containing 3 % of carbon sources, banana peel and corncob. After hydrolysis for 36 h, *A. awamori* NBRC 4388 produced reducing sugar from banana peel and corncob, 2.41 and 1.01 g/L as glucose equivalent, respectively. Successively, lactic acid fermentation by MPF with *A. awamori* NBRC 4388 and *L. mesenteroides* NBRC 3832 was performed for 48 h. This MPF using banana peel and corncob showed the highest d-lactic acid production, 0.88 and 0.31 g/L, respectively. *A. awamori* NBRC 4388 was thus used as the fungus of choice for MPF in the remainder of the study. After a 36 h incubation of *A. awamori* with banana peel and corncob biomass substrates, thin-layer chromatography (TLC) revealed that glucose and xylose were detectable as visible spots (data not shown). However, as the method used crude products, the spots detected by the TLC analysis were unclear and also appeared as other scattered spots. Based on these results, MPF was conducted by incubating *L. mesenteroides* with the *A. awamori* culture for 36 h.

### Effect of a biomass substrate pretreatment on d-lactic acid production

**Fig. 1:**
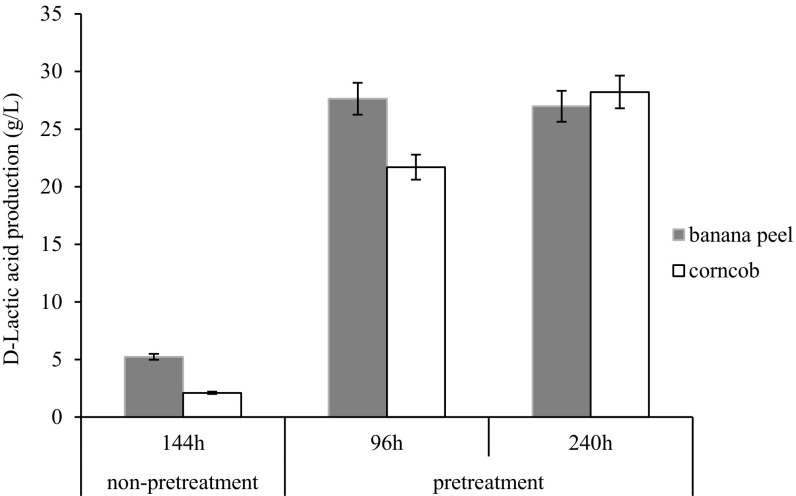
Effect of pretreatment of banana peel and corncob biomass substrates on d-lactic acid production. The results are expressed as d-lactic acid production (g/L) in multiple parallel fermentation using *A. awamori* NBRC 4388. The growth medium for cultivation is described in the text. After 36-h hydrolysis by *A. awamori* NBRC 4388, followed by inoculation of *L. mesenteroides* NBRC 3832 to d-lactic acid production by MPF, then cultivation was carried out with rotary shakers at 30 °C. The values represent the mean ± SD (*n* = 3)

In a preliminary experiment, the effect of a biomass substrate pretreatment on d-lactic acid production was examined. With banana peel and corncob biomass substrates, d-lactic acid production was higher with pretreatment than without pretreatment (Fig. 1). This result confirms that pretreatment for MPF is strongly recommended when using biomass substrate.

### d-Lactic acid production from biomass substrates by MPF

The carbon source noticeably affected MPF production of d-lactic acid. The effect, variance, and contribution of each selected factor were assessed by analysis of variance (ANOVA). Variables with a confidence level greater than 95 % were considered to have a significant influence over lactic acid production. The concentration of banana peel and corncob biomass substrates was of paramount importance for d-lactic acid production by MPF. The contributions of banana peel and corncob biomass substrates to d-lactic acid production were 97.20 and 56.06 %, respectively, and were supported by addition of KH_2_PO_4_, NaCl, Tween 80, yeast extract, MgSO_4_, and diammonium hydrogen citrate (Table 2). The results indicate that the type and concentration of the carbon source are the most important factors in determining optimal d-lactic acid production, and that each factor contributes differently to the production. Diammonium hydrogen citrate in MPF using banana peel and NaCl in MPF using corncob showed minor contributions to d-lactic acid production when these different sources of carbon were used as a substrate (0.043 and 0.044 %, respectively).

**Table 2:** Analysis of variance (ANOVA) for d-lactic acid production using banana peel (a) and corncob (b)

(a) Col/factors	DOF (f)	Sum of sqrs (S)	Variance (V)	F-ratio (F)	Pure sum (S’)	Percent P (%)
Banana peel	1	1575.693	1575.693	24428.458	1575.629	97.197
KH_2_PO_4_	1	11.599	11.599	179.838	11.535	0.711
NaCl	1	0.949	0.949	14.723	0.885	0.054
Tween 80	1	2.577	2.577	39.965	2.513	0.155
Yeast extract	1	2.775	2.775	43.03	2.711	0.167
MgSO_4_·7H_2_O	1	25.662	25.662	397.85	25.597	1.579
Diammonium hydrogen citrate	1	0.765	0.765	11.868	0.701	0.043
Error	16	1.031	0.064			0.094
Total	23	1621.056				100.00

(b) Col/factors	DOF (f)	Sum of sqrs (S)	Variance (V)	F-ratio (F)	Pure sum (S’)	Percent P (%)
Corncob	1	542.202	542.202	20823.353	542.176	56.060
KH_2_PO_4_	1	1.897	1.897	72.854	1.87	0.193
NaCl	1	0.455	0.455	17.499	0.429	0.044
Tween 80	1	118.691	118.691	4558.353	118.665	12.269
Yeast extract	1	109.731	109.731	4214.256	109.705	11.343
MgSO_4_·7H_2_O	1	109.32	109.32	4198.459	109.294	11.300
Diammonium hydrogen citrate	1	84.419	84.419	3242.148	84.393	8.726
Error	16	0.416	0.026			0.065
Total	23	967.135				100.00

#### Using banana peel as a substrate

Optimization of MPF revealed that production of d-lactic acid from banana peel substrate (31.8 g/L, productivity; 0.331 g/L/h) was most efficient with 15 % banana peel, 0.5 % KH_2_PO_4_, 0.1 % Tween 80, 0.05 % MgSO_4_·7H_2_O, 0.05 % NaCl, 1.5 % yeast extract, and 0.2 % diammonium hydrogen citrate using a rotary shaker after hydrolysis of the substrates by *A. awamori* for 36 h (Table 3a).

**Table 3:** Optimal condition and performance in validation of d-lactic acid production using banana peel (a) and corncob (b)

(a) Factors (%)	Level description	Level	Contribution (g/L)
Banana peel	15	2	8.102
KH_2_PO_4_	0.5	1	0.695
NaCl	0.05	1	0.198
Tween 80	0.1	2	0.327
Yeast extract	1.5	2	0.340
MgSO_4_·7H_2_O	0.05	1	1.034
Diammonium hydrogen citrate	0.2	1	0.178
Total contribution from all factors			10.870
Current grand average of performance			20.490
Expected result at optimum condition			31.364
Validation result			31.840

(b) Factors (%)	Level description	Level	Contribution (g/L)
Corncob	15	2	4.753
KH_2_PO_4_	0.5	1	0.281
NaCl	0.1	2	0.137
Tween 80	0.1	2	2.223
Yeast extract	1	1	2.138
MgSO_4_·7H_2_O	0.05	1	2.134
Diammonium hydrogen citrate	0.4	2	1.875
Total contribution from all factors			13.541
Current grand average of performance			23.739
Expected result at optimum condition			37.280
Validation result			38.260

All calculations and analysis were performed using Qualitek-4 software for automatic design

#### Using corncob as a substrate

Optimization of MPF revealed that production of d-lactic acid from corncob substrate (38.3 g/L, productivity; 0.159 g/L/h) was most efficient by MPF with 15 % corncob, 0.5 % KH_2_PO_4_, 0.1 % Tween 80, 0.05 % MgSO_4_·7H_2_O, 0.1 % NaCl, 1.0 % yeast extract, and 0.4 % diammonium hydrogen citrate using a rotary shaker after hydrolysis of substrates by *A. awamori* for 36 h (Table 3b).

The optimum fermentation time for high d-lactic acid production using banana peel and corncob were 96 and 240 h, respectively. d-Lactic acid production using banana peel required a much shorter fermentation time than that using corncob. l-Lactic acid was generally undetected (below 0.016 % of d-lactic acid); thus, the optical purity of d-lactic acid in MPFs using banana peel and corncob were calculated to be 99.99 and 99.98 %, respectively. d-Lactic acid production by MPF using banana peel is more efficient than using corncob. Specifically, d-lactic acid production by MPF using corncob (38.3 g/L productivity 0.159 g/L/h) exhibited higher yield than l-lactic acid by simultaneous saccharification fermentation of *Acremonium cellulose* and *Rhizopus* sp. using corncob (24.0 g/L productivity 0.170 g/L/h) (Miura et al. 2004). Thus, utilization of a biomass substrate as a carbon source has great potential for d-lactic acid production.

In MPF, carbon sources that *L. mesenteroides* can utilize for d-lactic acid fermentation are prepared by the enzymes produced by *A. awamori* and the productivity of d-lactic acid by *L. mesenteroides* is thought to be affected by the enzyme activities of *A. awamori* (data not shown). In this study, during hydrolytic processing when banana peel was used as the carbon source, β-glucosidase (6.28 U/mL) showed the highest enzymatic activity at 36 h, followed by endoglucanase (3.22 U/mL), amylase (2.34 U/mL), xylanase (0.31 U/mL), and pectinase (0.22 U/mL). β-Glucosidase (2.11 U/mL) and endoglucanase (1.01 U/mL) activities decreased on the second day after inoculation with *L. mesenteroides*, whereas amylase (5.23 U/mL) and xylanase (2.01 U/mL) activities increased until the second day after inoculation of *L. mesenteroides* (data not shown). When corncob was used as the carbon source, β-glucosidase showed the highest enzymatic activity (3.56 U/mL) at 36 h in the hydrolysis process, followed by amylase (2.87 U/mL), endoglucanase (0.98 U/mL), xylanase (0.87 U/mL), and pectinase (0.03 U/mL). After inoculation with *L. mesenteroides*, xylanase (2.01 U/mL) activity increased until the fourth day (data not shown). Banana peel and corncob are potential carbon sources for the production of β-glucosidase, amylase, endoglucanase and xylanase from *A. awamori* NBRC 4388.

Some previous productions of d-lactic acid from biomass have been reported. *L. plantarum* NCIMB 8826 produced 102.3 g/L of d-lactic acid from pulverized hardwood pulp by simultaneous saccharification and fermentation (Hama et al. 2015). *L. delbrueckii* LD 0025 and LD 0028 produced 62.6 g/L of d-lactic acid from 10 % rice saccharificate (Chang et al. 1999). Finally, *Escherichia coli* W3110 produced 48.7 g/L of d-lactic acid with 5.4 % molasses as the substrate (Shukla et al. 2004). Even though the d-lactic acid production from banana peel (31.8 g/L of d-lactic acid) and corncob (38.3 g/L of d-lactic acid) obtained in this study was not as high as that obtained from other carbon sources, our study indicated the high potential of these unutilized biomasses for d-lactic acid production in combination with *L. mesenteroides* and *A. awamori.*

*L. mesenteroides* is a heterofermentative microorganism that produces not only d-lactic acid, but also ethanol and acetic acid. In this study, in MPF using banana peel, 0.223 g/L of ethanol and 2.18 g/L of acetic acid were produced, respectively. In fermentation using corncob, 0.562 g/L of ethanol and 1.78 g/L of acetic acid were produced, respectively. These results showed that ethanol production using banana peel was a little lower than that using corncob and that acetic acid production using banana peel was a little higher than that using corncob.

## Conclusion

The Taguchi method using an L8 orthogonal array enabled us to analyze the influence of several factors and their interactions on d-lactic acid production using banana peel and corncob biomass substrates. Our data revealed that optimal d-lactic acid production using MPF from banana peel and corncob biomass substrates (31.8 g/L, productivity 0.331 g/L/h, optical purity 99.99 % and 38.3 g/L, productivity 0.159 g/L/h, optical purity 99.98 %, respectively) was achieved using 15 % carbon source (banana peel and corncob), 0.5 % KH_2_PO_4_, 0.1 % Tween 80, 0.05 % MgSO_4_·7H_2_O, 0.05 % (for banana peel) and 0.1 % (for corncob) NaCl, 1.5 % (for banana peel) and 1.0 % (for corncob) yeast extract, and 0.2 % (for banana peel) and 0.4 % (for corncob) diammonium hydrogen citrate. This result indicated that utilization of such biomass substrates in MPF has a high potential for d-lactic acid production.

## References

[CR1] Benninga H (1990). A history of lactic acid making.

[CR2] Carminati D, Giraffa G, Quiberoni A, Binetti A, Suarez V, Reinhemer J (2010) Advances and trends in starter cultures for dairy fermentation chapter 10: biotechnology of lactic acid bacteria: novel applications. Edited by Mozi F, Raya RR, Vignolo GM. Wiley Blackwell Publisher

[CR3] Chang DE, Jung HC, Rhee JS, Pan JG (1999). Homo fermentative production of d- or l-lactate in metabolically engineered *Escherichia coli* RR1. Appl Environ Microbiol.

[CR4] Coelho LF, de Lima CJ, Bernardo MP, Contiero J (2011). d-lactic acid production by *Leuconostoc mesenteroides* B512 using different carbon and nitrogen sources. Appl Biochem Biotechnol.

[CR5] Datta R, Tsai SP (1997). Lactic acid production and potential uses: a technology and economics assessment. ACS Symp Ser.

[CR6] Ghowdaman D, Ponnusami V (2015). Production and optimization of xylooligosaccharides from corncob by *Bacillus aerophilus* KGJ2 xylanase and its antioxidant potential. Int J Biol Macromol.

[CR7] Hama S, Mizuno S, Kihara M, Tanaka T, Ogino C, Noda H, Kondo A (2015). Production of d-lactic acid from hardwood pulp by mechanical milling followed by simultaneous saccharification and fermentation using metabolically engineered *Lactobacillus plantarum*. Bioresour Technol.

[CR8] Jeevan P, Nelson R, Rena E (2011). Optimization studies on acid hydrolysis of Corncob hemicellulosic hydrolysate for Microbial production of xylitol. J Microbiol Biotech Res.

[CR9] Lowry OH, Rosebrough NJ, Farr AL, Randall RJ (1951). Protein measurement with the folin phenol reagent. J Biol Chem.

[CR10] Lu H, Xiao Z, Yongze W, Xiaoren D, Jinhua W, Erin G, Ryan M, Andrew I, Shengde Z (2016). Enhancement of d-lactic acid production from a mixed glucose and xylose substrate by the *Escherichia coli* strain JH15 devoid of the glucose effect. BMC Biotechnol.

[CR11] Luo J, Xia L, Lin J, Cen P (1997). Kinetics of simultaneous saccharification and lactic acid fermentation processes. Biotechnol Prog.

[CR12] Miura S, Arimura T, Itoda N, Dwiarti L, Feng JB, Bin CH, Okabe M (2004). Production of l-lactic acid from corncob. J Biosci Bioeng.

[CR13] Ravella SR, Ganesh KC, Shetty PR, Phil JH (2008). The Taguchi methodology as a statistical tool for biotechnological applications: a critical appraisal. Biotechnol J.

[CR14] Rehman S, Hina A, Aqeel A, Shakeel AK, Muhammad S (2014). Production of plant cell wall degrading enzymes by monoculture and co-culture of *Aspergillus niger* and *Aspergillus terreus* under SSF of banana peel. Brazil J Microbiol.

[CR15] Richard FG, Nwabunma D (2010) Poly (lactic acid) synthesis, structures, properties, processing, and application. Wiley Series on Polymer Engineering and Technology, series editors. A John Wiley & Sons, Inc. Publication

[CR16] Ruengruglikit C, Hang YD (2003) L (+)-lactic acid production from corncobs by *Rhizopus oryzae* NRRL-395. Lebensm-Wiss. u.-Technol 36:573–575

[CR17] Sasaki C, Okumura R, Asakawa A, Asada C, Nakamura Y (2012) Production of d-lactic acid from sugarcane bagasse using steam-explosion. J Phys Conf Series 352(1)

[CR18] Shukla VB, Zhou S, Yomano LP, Shanmugam KT, Preston JF, Ingram LO (2004). Production of d-lactate from sucrose and molasses. Biotechnol Environ Lett.

[CR19] Somogyi M (1952). Notes on sugar determination. J Biol Chem.

[CR20] Tanaka T, Hoshina M, Tanabe S, Sakai K, Ohtsubo S, Taniguchi M (2005). Production of d-lactic acid from defatted rice bran by simultaneous saccharification and fermentation. Bioresour Technol.

[CR21] Wang YT, Xiang YF, Sang ML, Jie Y, Jian WL, Dong ZW, Yoon MK (2003). Purification of L (+)-lactic acid from fermentation broth with paper sludge as a cellulosic feedstock using weak anion exchanger Amberlite IRA-92. Biochem Eng J.

[CR22] Wang Q, Zhao X, Chamu J, Shanmugam KT (2011). Isolation, characterization and evolution of a new thermophilic *Bacillus licheniformis* for lactic acid production in mineral salts medium. Bioresour Technol.

[CR23] Zhou Y, Domínguez JM, Cao N, Du J, Tsao GT (1999) Optimization of L-lactic acid production from glucose by *Rhizopus oryzae* ATCC 52311. Appl Biochem Biotechnol Spring 77–79:401–7 10.1385/abab:78:1-3:401 15304710

